# Recuperative Hostels: Has the Law Been Violated?

**Published:** 1917-06-16

**Authors:** 


### RECUPERATIVE HOSTELS.

### Has the Law Been Violated?

We publish on another page two letters on this subject, one from Sir Frederick Milner, to whom all honour is due for the interest he has taken in our disabled soldiers and the work he has done for them; the other from Dr. Stewart Mackintosh. Both of these correspondents take objection to our article of May 19 on Recuperative Hostels. On reperusing this article, we have not a word to retract. If the hostels are really intended to receive men who are not mad, but are suffering from nervous or mental disease due to their experience in the field, we have nothing but recommendation and encouragement for the hostels, and commend them heartily to the support of our readers. We do not admit that we misrepresented Sir Frederick Milner's views as to the insane, and no word susceptible of such an interpretation can be found in the article. If it is a fact, as Sir Frederick Milner asserts, that numbers have been invalided from the Service, certified and sent to asylums, suffering not from madness, but from nerve-strain and exhaustion, then the law has been violated, and the Commissioners of the Board of Control have neglected their duty. Patients who have been so treated have their remedy at law, and the enforcement of this remedy in but one case would at once put a stop to the practice; and with the utmost respect to Sir Frederick Milner, we find it hard to believe that such a practice prevails.

If cases have already been received from asylums, and the receiving doctors testify that in the cases so received there are no signs of madness, it does not follow that there were no such signs when the patient was admitted into the asylum. He may have shown such signs, and have recovered sufficiently to permit of his discharge. The receipt by Sir Frederick Milner of letters from the inmates of asylums, saying that the writers are no more mad than he is, impresses us very little. Nothing is more frequent than for persons who are hopelessly insane to write such letters. No length of the list of those waiting to be received into hostels can prove that all the cases on the list are cases of sane men.

Sir Frederick Milner cannot object more than we do to a soldier who is not insane being sent to an asylum. Such a course is not only improper but illegal. But we say again that we shall not believe until proof is forthcoming that the Government has done this. Sir Frederick Milner says it is very easily proved, but he does not prove it. We are warmly sympathetic with his determination that justice shall be done to our injured soldiers; but we are not indifferent to justice being done to our officials. (*See also page* 209.)

